# Supplementary figures and images for: Spawning aggregation behavior and reproductive ecology of the giant bumphead parrotfish, Bolbometopon muricatum, in a remote marine reserve
Source: PeerJ. 2014 Nov 25;2:e681. doi: 10.7717/peerj.681 (PMC4250069; doi:10.7717/peerj.681)

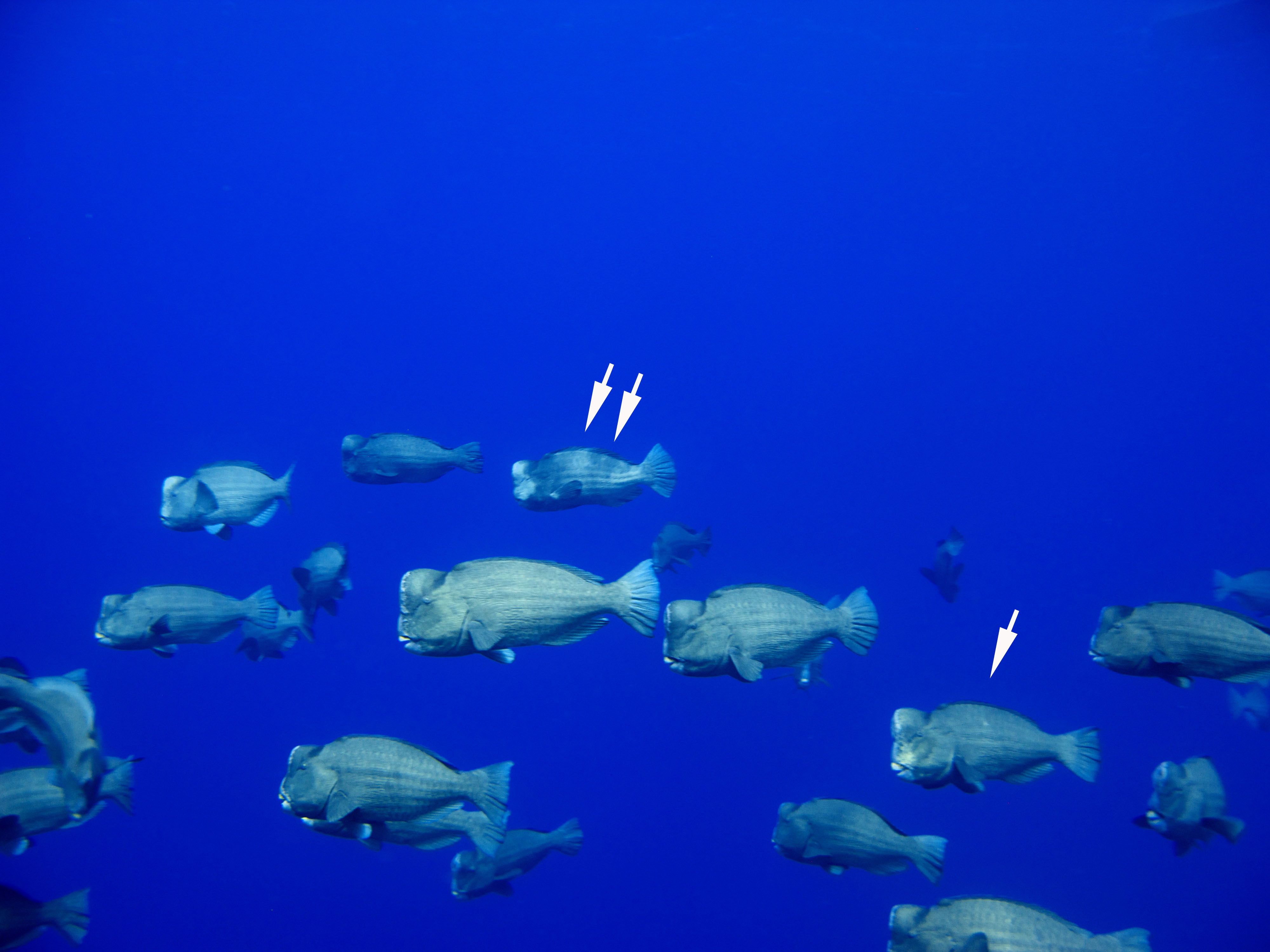

Supplement: Figure S1 — Note the male in lower right with a partially blanched face (single arrow), compared with the male in center background displaying fully blanched face, posterior caudal area, and bars (double arrow) apparently chasing one or both males in front of him. The recipients of this aggression can be seen displaying pale bars and partially blanched face. [file peerj-02-681-s001.jpg]

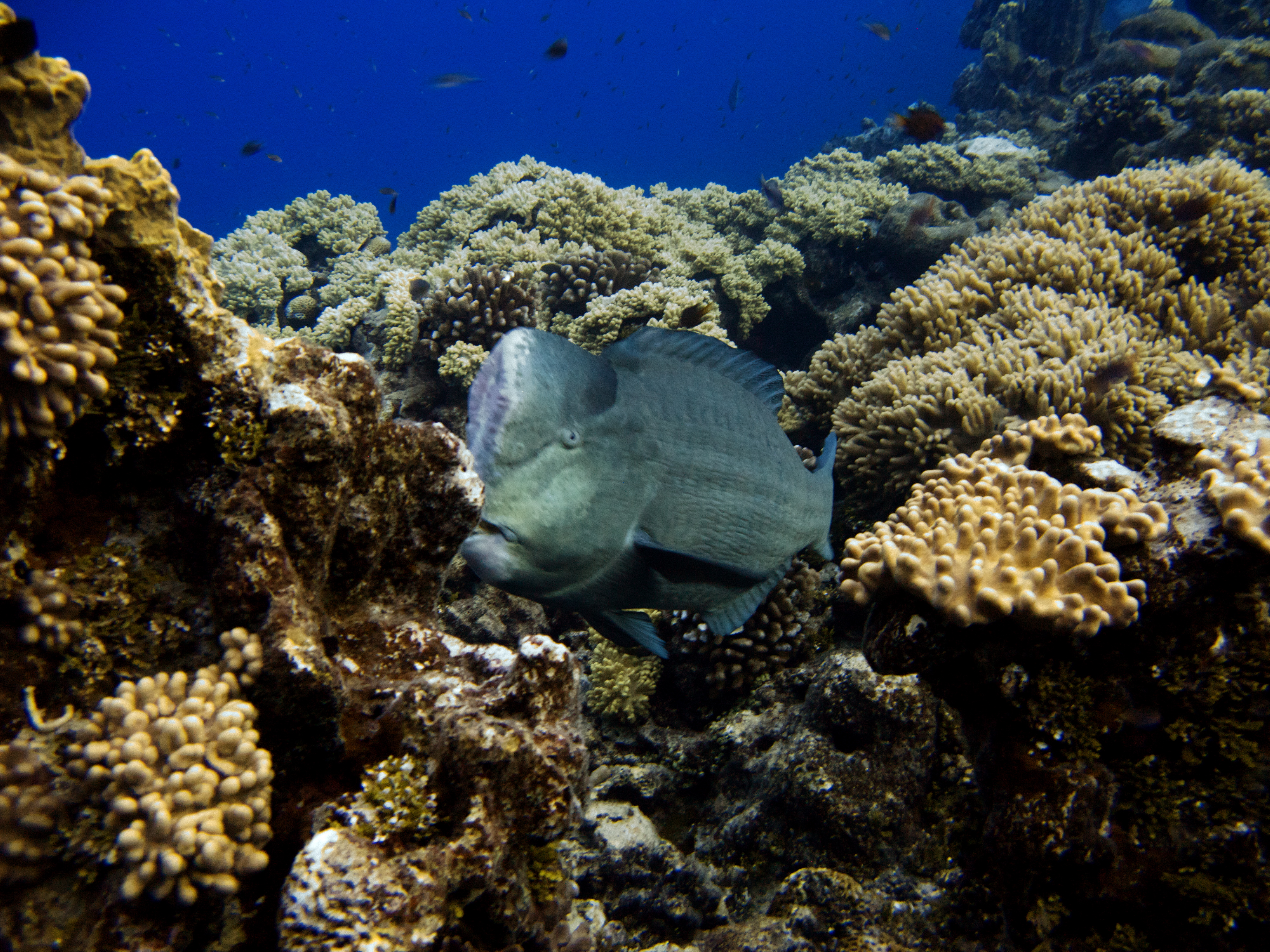

Supplement: Figure S2 — During the morning spawning period when stationary male Bolbometopon were not actively engaged in ascent/descent, courtship, or other behaviors, we observed these fish to occupy remarkably small (given their body size) areas of the benthos, as illustrated here. [file peerj-02-681-s002.jpg]
